# Recapitulation

**Published:** 1866-03

**Authors:** 


### RECAPITULATION.

Although the statements which have been made were presented in connection with the selection of the most healthful locality for building a new family residence, they are practically applicable to all cases wherein it may be desirable to make a house already built more comfortable and more healthful than it is, because, from what has been stated, it will be seen that a dwelling already erected should not be hastily and blindly abandoned merely on account of its insalubrity, for in the light of the above statements, it may be found that the causes of any present sickness are of a transient or of a remediable character, which may thus be illustrated:

The most favorable circumstances for the production of a miasmatic epidemic, speedy, malignant, and wide-spreading, are the exposure of the muddy bottom of a pond or sluggish stream to the beaming heat of a summer's sun. In less than a week whole neighborhoods have been stricken with disease, yet under such circumstances, and according to the well-established laws of miasm, five families may dwell within half a mile of a drained mill-pond, and yet only one will suffer from it, while the other four will remain exempt from unusual disease:

First. If a rapid stream half a mile wide runs between the drained pond and the house.

Second. If there is interposed a thick hedge or growth of living luxuriant trees or bushes. A treble row of sun-flowers are known to have answered the purpose in repeated cases.

Third. If the prevailing winds from June to October are from the house toward the pond.

Fourth. If the house be on a steep hill.

The reasons for the above exemptions are here shortly recapitulated:

First. Miasm does not cross a wide, rapid stream.

Second. Miasm is absorbed by thick, living luxuriant foliage.

Third. Miasm can not travel against the wind.

Fourth. Miasm can not ascend a high, steep hill.

There is no mystery in these variations, nor any complexity, when the laws of miasm are thoroughly understood.

It will be practically useful for the young farmer, in a pecuniary point of view, to understand further that in one year a house on the banks of a mill-pond or sluggish stream may be visited with sickness; the very next year that same house may be exempt, because it is a very cold summer; the third year it will escape, because it is a very hot summer; the fourth year it will be a very healthful habitation, because it has been a very wet summer. Why these variations?

First. Miasm can not form, or if it does, can not rise through a foot or two of depth of water, and the wet summer kept the bed of the pond covered.

Second. The hot summer dried the bed of the pond to dust, and there can be no miasm without dampness.

Third. The cold summer did not give the degree of heat necessary to the generation of miasm—that is, eighty degrees of Fahrenheit.

These principles fully explain the apparent mystery of the epidemics in New-Orleans, already referred to in the first part of this paper.

An illustration of the laws of miasm, which the reader will never forget, was had during a cholera summer in Boston, under the following circumstances: the city authorities inaugurated a most perfect system of cleanliness. Efforts were made to procure the services of the most reliable men to visit every house from cellar to garret, and compel the removal of every thing which could have even a remote tendency to invite the fearful scourge. The results were admirable; there was not a single case of cholera except in a very restricted district—in fact one family only was attacked. A more special examination was instituted, when there was found in a remote corner of the cellar a large pile of the accumulations of bad housekeeping for years; and this was in a state of putridity. On its removal, and the plentiful use of the most powerful disinfectants, the disease at once disappeared and did not return!

